# Adverse Outcomes in Patients on Chronic Anticoagulation Therapy Undergoing Sleeve Gastrectomy Conversions: Analysis of 30,664 Patients from the MBSAQIP Database

**DOI:** 10.1007/s11695-026-08635-z

**Published:** 2026-03-25

**Authors:** Juan S. Barajas-Gamboa, Gabriela Restrepo-Rodas, Valentin Mocanu, Mélissa V. Wills, Thomas H. Shin, Pattharasai Kachornvitaya, Gustavo Romero-Velez, Xinlei Zhu, Andrew T. Strong, Salvador Navarrete, Ricard Corcelles, A Daniel Guerron, John Rodriguez, Matthew Kroh, Jerry T. Dang

**Affiliations:** 1 Cleveland Clinic Abu Dhabi, Abu Dhabi, United Arab Emirates; 2 Khalifa University of Science and Technology, Abu Dhabi, United Arab Emirates; 3 Cleveland Clinic, Cleveland, USA; 4 University of Virginia Health System, Charlottesville, USA; 5 Cleveland Clinic Lerner College of Medicine, Cleveland, USA

**Keywords:** Chronic anticoagulation, sleeve gastrectomy, conversion, complications, bariatric surgery, MBSAQIP

## Abstract

**Introduction:**

The impact of chronic anticoagulation therapy (CAT) on sleeve gastrectomy (SG) conversions remains unclear. This study compared outcomes between patients with and without CAT undergoing SG conversions using the MBSAQIP database.

**Methods:**

A retrospective analysis of the MBSAQIP database (2020–2022) included patients undergoing conversion from SG to Roux-en-Y gastric bypass (RYGB), single anastomosis duodeno-ileal bypass (SADI), or biliopancreatic diversion with duodenal switch (BPD-DS). Primary outcomes were 30-day serious complications. Secondary outcomes included specific complications, length of stay, and operative time. Multivariable logistic regression assessed whether CAT independently predicted outcomes.

**Results:**

Of 30,664 patients, 935 (3.05%) were on CAT. CAT patients were older (52.08 vs 45.73 years), had higher BMI (42.34 vs 40.33 kg/m²), and higher rates of diabetes (24.71% vs 11.55%) and hypertension (82.21% vs71.32%) (all p<0.001). Primary conversion indications were reflux disease(51.45%), weight recurrence (26.40%) and insufficient weight loss (14.86%).CAT patients had higher rates of serious complications (14.12% vs 6.42%),readmission (13.80% vs 6.88%), reoperation (4.92% vs 2.77%), and bleeding(4.81% vs 1.68%), with longer operative times (158.63 vs 144.01 minutes)and hospital stays (2.27 vs 1.68 days) (all p<0.001). On multivariable analysis, CAT was not independently associated with serious complications(OR 1.06, 95%CI 0.61-1.85, p=0.824).

**Conclusion:**

Patients on CAT undergoing SG conversions experience higher complication rates and longer hospital stays; However, anticoagulation therapy is not independently associated with increased serious complication safter adjusting for obesity-related conditions, highlighting the importance of comprehensive risk assessment and optimization of underlying medical conditions in all high-risk patients undergoing conversion bariatric surgery.

## Key Points

• Patients on chronic anticoagulation undergoing sleeve conversions had higher complication rates (14.1% vs. 6.4%), primarily driven by their obesity-related conditions burden.

• Anticoagulation was not independently associated with complications (OR 1.06).

• Bleeding complications were more frequent in anticoagulated patients (4.8% vs. 1.6%, *p* < 0.001), warranting enhanced perioperative monitoring.

• Underlying obesity-related conditions, rather than anticoagulation alone, are the primary drivers of increased perioperative risk.

## Introduction

Sleeve gastrectomy (SG) is the most commonly performed metabolic and bariatric surgery (MBS) in the United States, accounting for 60.4% of all bariatric procedures in 2023 [1]. Despite its increasing popularity as an effective treatment for obesity, a subset of patients- particularly those with multiple obesity-related conditions such as cardiovascular disease, diabetes, and sleep apnea- may eventually require conversion to alternative bariatric procedures due to recurrence of weight, insufficient weight loss, complications, or persistent metabolic issues that fail to resolve with initial surgery [2, 3]. 

In conversion bariatric surgery populations, approximately 3% of patients are on CAT for previous venous thromboembolism, pulmonary embolism, or atrial fibrillation [4, 5]. This prevalence may be influenced by the study period (2020–2022), which coincided with the COVID-19 pandemic when heightened thrombotic awareness led to increased anticoagulation use [6]. These challenges become even more complex following MBS, as altered gastrointestinal anatomy and physiology can impact anticoagulant efficacy [7]. Studies have shown that changes in gastric pH, accelerated transit time, and reduced absorptive surfaces may impair anticoagulant absorption [8, 9]. The mechanism of impaired anticoagulant absorption varies by procedure type and medication class. In SG, the primary mechanisms include reduced gastric surface area and altered gastric pH, which particularly affects vitamin K antagonists like warfarin [8]. For direct oral anticoagulants (DOACs), SG has less impact on absorption compared to procedures with malabsorptive components like Roux-en-Y gastric bypass (RYGB), where bypassing the jejunum can lead to unpredictable drug levels, as it is the primary absorption site for rivaroxaban and apixaban [10]. Previous studies demonstrated that while SG patients may require minor warfarin dosage adjustments, RYGB patients often need substantial dose increases of 20–50% to maintain therapeutic INR levels [4, 8, 11]. 

The impact of CAT on bariatric surgery outcomes has been explored in both single-center and registry-based studies. Fares et al. found no significant differences in postoperative outcomes between patients with and without CAT undergoing primary bariatric surgery, suggesting that MBS is safe for this high-risk population [12]. In contrast, a study based on the Metabolic and Bariatric Surgery Accreditation and Quality Improvement Program (MBSAQIP) database, analyzing patients undergoing primary adjustable gastric banding (AGB), SG, and RYGB reported significantly worse postoperative outcomes in patients on preoperative anticoagulation [13]. However, data specifically addressing the impact of CAT on revisional bariatric surgery, particularly SG conversions, remain limited. Given that conversional bariatric surgery is associated with higher complication rates compared to primary procedures, it is crucial to understand how CAT influences postoperative outcomes in this setting [3, 14, 15]. 

Notably, conversional bariatric surgery has gained significant traction in recent years, though exact epidemiological data is scarce [2, 3]. Estimates suggest that 18–36% of SG patients undergo conversion to another bariatric procedure [2, 16]. As the first wave of SG patients from the early 2010s reaches the decade mark post-procedure, a substantial proportion (estimated at 25–35%) now requires conversion for insufficient weight loss, refractory GERD, or other complications [16]. Common conversion procedures include RYGB for GERD or inadequate weight loss, duodenal switch (DS) for severe obesity or metabolic disease, and single anastomosis duodeno-ileal bypass (SADI) for insufficient weight loss [2, 17, 18]. This growing population represents a unique surgical challenge, as these patients often present with more complex anatomy, increased scar tissue, and higher rates of obesity-related conditions including conditions requiring chronic anticoagulation than primary bariatric surgery candidates. Understanding the specific risks faced by anticoagulated patients in this increasingly common clinical scenario is therefore of paramount importance.

Given this significant knowledge gap in this increasingly prevalent clinical scenario, this study aims to compare the perioperative outcomes of patients with and without CAT undergoing SG conversion to RYGB, SADI, or biliopancreatic diversion with duodenal switch (BPD-DS) using the MBSAQIP database.

## Materials and Methods

### Study Design and Ethical Approvals:

This is a retrospective cohort study utilizing prospectively collected data. The study was deemed exempt from Institutional Review Board (IRB) approval due to the use of de-identified data from a national database.

### Data Source:

 Data for this study was obtained from the 2020–2022 MBSAQIP database. This period was selected due to the inclusion of granular data on conversion bariatric procedures. The MBSAQIP database collects preoperative, intraoperative, and postoperative data from approximately 900 accredited bariatric centers across the United States and Canada, ensuring a comprehensive dataset.

### Study Population:

Patients undergoing SG conversion to RYGB, SADI, or BPD-DS between 2020 and 2022 were included in the analysis. The variables “conv_prev_proc” and “conv_curr_proc” were used to identify the procedure types. Patients were then categorized based on the presence or absence of CAT at the time of surgery.

### Inclusion and Exclusion Criteria:

Inclusion criteria encompassed all patients aged 18 years or older who underwent SG conversion to RYGB, SADI, or BPD-DS within the study period. Exclusion criteria included patients who underwent primary bariatric surgery, non-conversion revisional procedures, reversal surgeries, emergency bariatric procedures, concurrent surgical procedures, or surgeries performed for non-bariatric indications. Cases with missing key data were excluded from the study.

### Definitions:

- **Conversion** **surgery **was defined as any subsequent bariatric procedure altering the original anatomical structure of the primary operation to enhance weight loss or resolve complications.
- **CAT (Chronic Anticoagulation Therapy)** was defined as continuous use of anticoagulants for conditions such as atrial fibrillation, venous thromboembolism, or mechanical heart valves. The MBSAQIP only collects data on whether the patient is or is not on CAT but not the specific regimen or medications utilized.
- **Serious complications** were defined as a composite outcome comprising any occurrence of the following within 30 days postoperatively: anastomotic leak, postoperative bleeding, reoperation, non-operative intervention, cardiac arrest, myocardial infarction, cardiopulmonary resuscitation, pneumonia, unplanned intubation, acute kidney injury, venous thromboembolism, deep surgical site infection, sepsis, or cerebrovascular accident.

### Patient Variables:

Basic demographic data such as age, sex, race, and BMI were collected. Within 30 days postoperatively, multiple variables were assessed including: patient obesity-related conditions such as diabetes (categorized as non-diabetic, diet-controlled, non-insulin-dependent, and insulin-dependent), hypertension, GERD, COPD, hyperlipidemia, chronic steroid use, renal insufficiency, dialysis dependence, venous stasis, preoperative therapeutic anticoagulant use, and obstructive sleep apnea; prior medical events including VTE, MI, percutaneous coronary intervention, and major cardiac surgery; and functional capacity measurements through preoperative functional status (classified as independent, partially dependent, or fully dependent) and ASA Physical Status classification.

### Outcome Variables:

Primary outcomes included indications for conversion surgery and 30-day serious complications. Secondary outcomes encompassed specific postoperative complications, length of hospital stay, and operative time. Additional outcomes assessed included rates of readmission, reoperation, and need for surgical interventions within 30 days postoperatively.

### Statistical Analysis:

Statistical analysis was performed using STATA 15 (StataCorp, College Station, TX, USA). Categorical variables were expressed as percentages, while continuous variables were reported as means ± standard deviations. Chi-square tests were used for categorical comparisons, and independent t-tests were utilized for continuous variables. Despite the significant sample size difference between groups (CAT: *n* = 935 vs. non-CAT: *n* = 29,729), these statistical methods remain valid for unbalanced designs and provide appropriate comparison of outcomes. Multivariable logistic regression was conducted to assess whether CAT was independently associated with postoperative outcomes, adjusting for potential confounders such as age, BMI, and obesity-related conditions. This regression approach is specifically designed to handle unbalanced groups and accounts for baseline differences between cohorts, allowing isolation of the independent effect of CAT on outcomes. Variables with a p-value < 0.1 in univariate analysis were included in the multivariable model. Statistical significance was defined as *p* < 0.05.

## Results

### Patient Demographics and Clinical Characteristics

Of the 30,664 patients included, 935 (3.05%) were on CAT at the time of conversion surgery. As expected, CAT patients had a higher burden of cardiovascular and metabolic conditions. CAT patients were older (52.08 ± 11.42 vs. 45.73 ± 11.06 years), had higher BMI (42.34 ± 7.82 vs. 40.33 ± 6.65 kg/m²), and were more likely to be male (19.25% vs. 9.55%).

The CAT group demonstrated higher prevalence of obesity-related conditions, including diabetes mellitus (24.7% vs. 11.6%), with more patients classified as insulin-dependent (15.5% vs. 9.0%), hypertension (82.2% vs. 71.3%), hyperlipidemia (38.8% vs. 21.0%), and obstructive sleep apnea (67.4% vs. 59.5%). Consistent with indications for chronic anticoagulation, the CAT group had substantially higher rates of prior venous thromboembolism (13.9% vs. 0.6%), myocardial infarction (2.9% vs. 0.3%), percutaneous coronary intervention (3.2% vs. 0.2%), and major cardiac surgery (1.6% vs. 0.1%). The CAT group had a higher proportion of ASA class 3 patients (87.9% vs. 77.0%). Detailed baseline characteristics are presented in Table 1.

**Table 1:** Patient demographics*

Variable	CAT (*N* = 935)	Non-CAT (*N* = 29,729)	*P*-value
Demographics			
Age, Mean (SD)	52.08 (10.6)	45.73 (10.3)	
Male, N (%)	180 (19.2)	2,839 (9.5)	
BMI, Mean (SD)	42.3 (9.6)	40.3 (8)	*p* < 0.001
ASA Class, N (%)			
ASA I, N (%)	1 (0.1)	55 (0.1)	
ASA II, N (%)	97 (10.3)	7,817 (26.2)	
ASA III, N (%)	767 (82)	21,181 (71.2)	
ASA IV, N (%)	67 (7.1)	644 (2.1)	
Comorbidities, N (%)			
Smoker in previous year	53 (5.6)	1,375 (4.6)	0.136
Diabetes Mellitus type 2			
None or diet controlled	704 (75.2)	26,296 (88.4)	
Non-insulin dependent	86 (9.2)	793 (2.6)	
Insulin dependent	145 (15.5)	2,640 (8.8)	
Hypertension	137 (61.8)	2,283 (33.9)	
Gastroesophageal reflux disease	698 (74.6)	20,758 (69.8)	0.002
Chronic obstructive pulmonary disease	39 (4.1)	311 (1)	*p* < 0.001
Hyperlipidemia	411 (43.9)	5,037 (16.9)	
Chronic steroid use	16 (7.2)	167 (2.4)	*p* < 0.001
Chronic kidney disease	14 (1.5)	95 (0.3)	*p* < 0.001
Dialysis dependent	8 (0.8)	45 (0.1)	
History of venous thromboembolism	432 (46.2)	652 (2.1)	
Venous Stasis	43 (4.6)	114 (0.3)	
Obstructive sleep apnea	416 (44.4)	7,052 (23.7)	*p* < 0.001
History of myocardial infarction	82 (8.7)	232 (0.7)	
Percutaneous coronary intervention	65 (6.9)	169 (0.5)	
Previous major cardiac surgery	124 (13.2)	264 (0.89)	

*CAT *Chronic Anticoagulation Therapy, *Non-CAT *Not onChronic Anticoagulation Therapy, *ASA *American Society of Anesthesiologists, *SD *Standard Deviation

* Baseline characteristics are presented for descriptive purposes. As expected, the CAT group had higher burden of cardiovascular and metabolic conditions that necessitate anticoagulation therapy

### Perioperative Outcomes and Postoperative Complications

The most common conversion procedure in both groups was SG to RYGB, occurring in 86.1% of CAT cases and 89.4% of non-CAT cases (*p* = 0.005). Conversions to SADI and BPD-DS were more frequent in the CAT group (5.4% vs. 4.3%, *p* = 0.005, and 8.4% vs. 6.2%, *p* = 0.005, respectively). GERD was the leading indication for conversion in both groups, though it was slightly less frequent in the CAT group (44.5% vs. 51.6%, *p* < 0.001). Conversely, the CAT group had a higher proportion of conversions due to suboptimal weight loss (26.0% vs. 14.7%, *p* < 0.001) but fewer due to recurrence of weight (17.5% vs. 26.4%, *p* < 0.001).

The majority of procedures in both groups were performed laparoscopically, though open surgery was significantly higher in the CAT group (1.1% vs. 0.4%, *p* < 0.001). The mean operative time was significantly longer in the CAT group (158 ± 74 vs. 144 ± 66 min, *p* < 0.001). Similarly, mean length of hospital stay was significantly longer in the CAT group (2.27 ± 2.4 vs. 1.68 ± 1.7 days, *p* < 0.001).

The overall rate of serious complications was significantly higher in the CAT group (14.1% vs. 6.4%, *p* < 0.001). Postoperative bleeding occurred more frequently in the CAT group (4.8% vs. 1.6%, *p* < 0.001), as did the need for reoperation (4.9% vs. 2.7%, *p* < 0.001) and non-operative interventions (5.1% vs. 2.1%, *p* < 0.001). Readmission rates were also nearly double in the CAT group (13.8% vs. 6.8%, *p* < 0.001). Cardiac events (0.6% vs. 0.08%, *p* < 0.001), pneumonia (1.1% vs. 0.4%, *p* < 0.001), acute kidney injury (0.5% vs. 0.07%, *p* < 0.001), and sepsis (0.8% vs. 0.2%, *p* < 0.001) were significantly more common in the CAT group. Additionally, gastrointestinal tract bleeding (2.4% vs. 0.6%, *p* < 0.001) and deep surgical site infections (2.0% vs. 1.05%, *p* = 0.004) occurred at higher rates. There was no significant difference in venous thromboembolism (0.4% vs. 0.3%, *p* = 0.566), though there was a significant difference in pulmonary embolism between groups (0.11 vs. 0.17%, *p* < 0.001). Detailed perioperative and postoperative outcomes are presented in Table 2.

**Table 2:** Perioperative and postoperative outcomes

Variable	CAT (*N* = 935)	Non-CAT (*N* = 29,729)	*P*-value
Conversions			
SG to RYGB, N (%)	805 (86.1)	26,583 (89.4)	*p* = 0.005
SG to SADI, N (%)	51 (5.4)	1,297 (4.3)	*p* = 0.005
SG to BPD-DS, N (%)	79 (8.4)	1,849 (6.2)	*p* = 0.005
Surgical approach			
Endoscopic, N (%)	1 (0.1)	6 (0.02)	*p* < 0.001
Laparoscopic, N (%)	923 (98.7)	29,604 (99.5)	*p* < 0.001
Open, N (%)	11 (1.1)	119 (0.4)	*p* < 0.001
Indication			
GERD, N (%)	417 (44.5)	15,361 (51.6)	*p* < 0.001
Weight gain, N (%)	244 (17.5)	7,850 (26.4)	*p* < 0.001
Suboptimal weight loss, N (%)	164 (26)	4,393 (14.7)	*p* < 0.001
Mean operative time, mean (SD)	158 (74)	144 (66)	*p* < 0.001
Length of stay days, mean (SD)	2.27 (2.4)	1.68 (1.7)	*p* < 0.001
Postoperative complications			
Anastomotic Leak, N (%)	10 (1.0)	179 (0.6)	0.07
Postoperative bleeding, N (%)	45 (4.8)	498 (1.6)	*p* < 0.001
Reoperation, N (%)	46 (4.9)	823 (2.7)	*p* < 0.001
Non-operative intervention, N (%)	48 (5.1)	627 (2.1)	*p* < 0.001
Readmission, N (%)	129 (13.8)	2,046 (6.8)	*p* < 0.001
Cardiac Events, N (%)	6 (0.6)	24 (0.08)	*p* < 0.001
Pneumonia, N (%)	11 (1.1)	120 (0.4)	*p* < 0.001
Pulmonary embolism, N (%)	1 (0.11)	51 (0.17)	*p* < 0.001
Venous thromboembolism, N (%)	4 (0.4)	95 (0.3)	0.566
Acute kidney injury, N (%)	5 (0.5)	20 (0.07)	*p* < 0.001
GI Tract Bleed, N (%)	23 (2.4)	199 (0.6)	*p* < 0.001
Deep surgical site infection, N (%)	19 (2.0)	311 (1.05)	*p* = 0.004
Sepsis, N (%)	8 (0.8)	70 (0.2)	*p* < 0.001
Serious complications, N (%)	132 (14.1)	1,909 (6.4)	*p* < 0.001

*CAT *Chronic Anticoagulation Therapy, *Non-CAT *Not on Chronic Anticoagulation Therapy, *SG *Sleeve Gastrectomy, *RYGB *Roux en Y Gastric Bypass, *SADI *Single anastomosis duodeno-ileal bypass, *BPD/DS *Biliopancreatic diversion with duodenal switch, *GERD *GastroesophagealReflux Disease

### Multivariate Analysis for Serious Complications

After adjusting for patient factors and operative characteristics in multivariable logistic regression analysis, CAT was not independently associated with an increased risk of serious complications (OR 1.0645, 95% CI 0.6126-1.8500, *p* = 0.824). This finding contrasts with the significant differences observed in univariate analysis, suggesting that the higher complication rates in CAT patients are likely attributable to their greater burden of obesity-related conditions rather than anticoagulation therapy alone. Significant independent predictors of complications included longer operative time (OR 1.0026 per minute, 95% CI 1.0014–1.0039, *p* < 0.001) and a history of venous thromboembolism (OR 1.6543, 95% CI 1.0177–2.6891, *p* = 0.042). Other variables, including age (OR 0.9617 per 10 years, 95% CI 0.8645–1.0698, *p* = 0.473), hypertension (OR 1.1907, 95% CI 0.9485–1.4948, *p* = 0.132), hyperlipidemia (OR 1.1285, 95% CI 0.8546–1.4902, *p* = 0.394), and smoking (OR 0.8978, 95% CI 0.5539–1.4554, *p* = 0.662), were not independently associated with complications. The multivariable model demonstrated modest discriminative ability (area under the receiver operating characteristic curve = 0.61) and calibration (Brier score = 0.07). Detailed characteristics analyzed are presented in Table 3.

**Table 3:** Multivariate analysis for serious complications

Risk Factor	Adjusted Odds Ratio*	95% Confidence Interval	*P*-value
Age (per 10 years)	0.9617	0.8645–1.0698	0.473
BMI (Category 5)	0.8325	0.7764–0.8928	< 0.001
Therapeutic Anticoagulation	1.0645	0.6126–1.8500	0.824
Hyperlipidemia	1.1285	0.8546–1.4902	0.85
History of miocardial infarction	1.6778	0.7531–3.7376	0.205
Operative length	1.0026	1.0014–1.0039	< 0.001
Hipertension	1.1907	0.9485–1.4948	0.132
Smoker	0.8978	0.5539–1.4554	0.662
History of venous thrombosis	1.6543	1.0177–2.6891	0.042
Chronic obstructive pulmonary disease	1.3905	0.6419–3.0122	0.403
SG2SADI vs. SG2RYGB†	0.9029	0.5334–1.5285	0.704
SG2BPD vs. SG2RYGB†	0.9750	0.6230–1.5258	0.912
Albumin (per unit)	0.8643	0.6824–1.0948	0.227

*BMI *Body Mass Index, *SG2RYGB *Sleeve Gastrectomy to Roux-en-Y Gastric Bypass, *SG2SADI *Sleeve Gastrectomy to Single Anastomosis Duodeno-Ileal Bypass, *SG2BPD *Sleeve Gastrectomy to Biliopancreatic Diversion

*Model adjusted for age, gender, BMI, and comorbidities. Area under the receiver operating characteristic curve = 0.61, Brier score = 0.07

†Reference category is SG2RYGB (Sleeve Gastrectomy to Roux-en-Y Gastric Bypass)

### Multivariate Analysis for Length of Stay

After multivariable adjustment, significant independent predictors of increased length of stay included therapeutic anticoagulation (OR 1.7167, 95% CI 1.1349–2.5968, *p* = 0.010), hypertension (OR 1.3292, 95% CI 1.1146–1.5852, *p* = 0.002), history of venous thrombosis (OR 1.5696, 95% CI 1.0618–2.3202, *p* = 0.024), chronic obstructive pulmonary disease (OR 2.1671, 95% CI 1.2166–3.8602, *p* = 0.009), and longer operative time (OR 1.0068 per minute, 95% CI 1.0058–1.0078, *p* < 0.001). Higher albumin levels were protective (OR 0.6564 per unit, 95% CI 0.5436–0.7926, *p* < 0.001). Compared to SG conversion to RYGB (SG2RYGB), SG conversion to SADI (SG2SADI) was associated with shorter length of stay (OR 0.5929, 95% CI 0.3772–0.9320, *p* = 0.023), while SG conversion to BPD-DS (SG2BPD) was associated with longer length of stay (OR 1.4070, 95% CI 1.0419–1.8999, *p* = 0.026). Other factors were not independently associated with length of stay. The model demonstrated moderate discriminative ability (AUC = 0.67) and calibration (Brier score = 0.12). Detailed characteristics are presented in Table 4.

**Table 4:** Multivariate analysis for length of stay

Risk Factor	Adjusted Odds Ratio*	95% Confidence Interval	*P*-value
Age (per 10 years)	0.9806	0.9019–1.0662	0.647
BMI (Category 5)	0.8987	0.8531–0.9467	< 0.001
Therapeutic anticoagulation	1.7167	1.1349–2.5968	0.010
Hyperlipidemia	1.1341	0.9131–1.4087	0.255
History of myocardial infarction	0.8370	0.3899–1.7968	0.648
Operative length	1.0068	1.0058–1.0078	< 0.001
Hypertension	1.3292	1.1146–1.5852	0.002
Smoker	1.1583	0.8150–1.6462	0.413
History of venous thrombosis	1.5696	1.0618–2.3202	0.024
Chronic obstructive pulmonary disease	2.1671	1.2166–3.8602	0.009
SG2SADI vs. SG2RYGB†	0.5929	0.3772–0.9320	0.023
SG2BPD vs. SG2RYGB†	1.4070	1.0419–1.8999	0.026
Albumin (per unit)	0.6564	0.5436–0.7926	< 0.001

*BMI *Body Mass Index, *SG2RYGB *Sleeve Gastrectomy to Roux-en-Y Gastric Bypass, *SG2SADI *Sleeve Gastrectomy to Single Anastomosis Duodeno-Ileal Bypass, *SG2BPD *Sleeve Gastrectomy to Biliopancreatic Diversion

*Model adjusted for age, gender, BMI, and comorbidities. Area under the receiver operating characteristic curve = 0.67, Brier score = 0.12

†Reference category is SG2RYGB (Sleeve Gastrectomy to Roux-en-Y Gastric Bypass)

### Outcomes by Conversion Procedure

Detailed postoperative outcomes by conversion procedure type are presented in Table 5. SG2RYGB had a significantly higher rate of serious complications (6.8% in SG2RYGB vs. 4.4% in SG2SADI vs. 4.8% in SG2BPD, *p* < 0.001), as well as higher rates bleeding (1.8% in SG2RYGB vs. 0.6% in SG2SADI vs. 0.9% in SG2BPD, *p* < 0.001 and gastrointestinal tract bleeding (0.7% in SG2RYGB vs. 0.07% in SG2SADI vs. 0.6% in SG2BPD, p 0.01). Conversely, SG2SADI had significantly higher rates of anastomotic leaks (1.2% in SG2SADI vs. 0.5% SG2RYGB vs. 0.8% SG2BPD, *p* = 0.02.

**Table 5:** Outcomes by conversion procedure type

Outcome	SG2RYGB (*n* = 27,388)	SG2SADI (*n* = 1,348)	SG2BPD (*n* = 1,928)	*p*-value
Operative Characteristics				
Operative time, mean (SD), min	144.5 (66.9)	144.1 (64.3)	143.9 (65)	0.916
Hospital length of stay, mean (SD), days	1.7 (1.8)	1.5 (1.6)	1.6 (1.4)	0.03
Postoperative Complications				
Serious complications, n (%)	1,885 (6.8)	60 (4.4)	96 (4.8)	< 0.001
Anastomotic Leaks, n (%)	155 (0.5)	17 (1.2)	17 (0.8)	0.02
Bleeding, n (%)	515 (1.8)	9 (0.6)	19 (0.9)	< 0.001
Gastrointestinal tract bleeding, n (%)	209 (0.7)	1 (0.07)	12 (0.6)	0.01
Deep surgical site infection, n (%)	282 (1.03)	22 (1.63)	26 (1.35)	0.05
Readmission, n (%)	2,009 (7.3)	63 (4.6)	103 (5.3)	< 0.001
Reoperation, n (%)	790 (2.8)	34 (2.5)	45 (2.3)	0.28
Non-operative intervention, n (%)	629 (2.3)	17 (1.2)	29 (1.5)	0.004
Venous thromboembolism, n (%)	91 (0.3)	3 (0.2)	5 (0.2)	0.69
Pulmonary embolism, n (%)	48 (0.1)	0 (0)	4 (0.2)	0.28
Sepsis, n (%)	70 (0.2)	1 (0.07)	7 (0.3)	0.26

*SG2RYGB *Sleeve Gastrectomy to Roux-en-Y Gastric Bypass, *SG2SADI *Sleeve Gastrectomy to Single Anastomosis Duodeno-Ileal Bypass,* SG2BPD* Sleeve Gastrectomy to Biliopancreatic Diversion, *SD *Standard Deviation

## Discussion

CAT presents unique challenges in bariatric surgery, particularly when converting patients with previous SG to other bariatric procedures. This study emphasizes the clinical importance of recognizing and managing perioperative risks for patients on CAT who undergo conversion bariatric procedures. Our analysis revealed significant demographic and clinical differences between the groups. Patients in the CAT group were significantly older (52 vs. 45, *p* < 0.001) and had a higher prevalence of obesity-related conditions. The CAT group experienced significantly longer operative times and increased length of hospital stay. This group also had nearly doubled readmission rates (13.8% vs. 6.8%, *p* < 0.001), with higher rates of serious complications (14.1% vs. 6.4%, *p* < 0.001), postoperative bleeding (4.8% vs. 1.6%, *p* < 0.001), and reoperation (4.9% vs. 2.7%, *p* < 0.001. However, the multivariable analysis revealed that CAT was not independently associated with increased complications (OR 1.0645, 95% CI 0.6126-1.8500, *p* = 0.824).

The impact of bariatric surgery on anticoagulation drug efficacy remains a concern, particularly in conversion procedures that involve greater anatomical modification [19, 20]. Prior studies have noted transient alterations in warfarin response and some variability in DOAC absorption, though most patients maintain therapeutic levels postoperatively [8–11, 19]. Patients requiring CAT represent a high-risk cohort due to their burden of obesity-related conditions, a finding corroborated by previous studies [7, 12]. While several studies have investigated the outcomes of CAT in primary bariatric procedures, deeming it safe in patients with CAT, there remains a significant knowledge gap regarding conversion procedures [21], which typically involve greater technical complexity and higher baseline complication rates, irrespective of anticoagulation status [6, 8, 22, 23]. These procedures often involve anatomical alterations such as trimming of the pouch or creation of different anastomoses, which may alter the bioavailability of these drugs [8–10]. 

Intraoperatively, CAT patients had longer operative times and hospital stays, consistent with the added technical challenges of revisional bariatric surgery. Fares et al. found no significant difference in operative time in primary cases (126 min in CAT vs. 122 min in non-CAT, *p* = 0.68), suggesting that this effect is more pronounced in conversions ((158 vs. 144 min, *p* < 0.001) [12]. The increased rate of conversion to open surgery in the CAT group, though small in absolute numbers, supports this notion, as anticoagulation may introduce technical challenges necessitating conversion. Surgeons should consider strategies such as early use of additional trocars, meticulous hemostasis with advanced energy devices, and lower thresholds for vascular surgery consultation in complex cases. Moreover, since conversion procedures require more extensive dissection and adhesiolysis, this subgroup warrants careful intraoperative planning [2, 24, 25]. Fares et al. reported a prolonged stay (4.6 days in CAT vs. 2.6 days in non-CAT, *p* < 0.00), likely due to the need for careful postoperative anticoagulation monitoring and management, which is comparable to our findings (2.27 days in CAT vs. 1.68 days in non-CAT, *p* < 0.001).

Postoperatively, CAT patients experienced higher rates of serious complications, including bleeding, reoperation, and readmission, consistent with prior studies demonstrating increased bleeding risks in anticoagulated patients undergoing major surgery [7, 13, 22]. Altieri et al. reported higher bleeding rates (OR 2.7, *p* < 0.0001) and increased reoperation and readmission risks in primary bariatric surgery, findings that our study extends to conversion procedures [13]. Furthermore, Sebastian et al. noted higher transfusion needs (1.7% vs. 0.7%, *p* < 0.001) and gastrointestinal bleeding (1.2% vs. 0.5%, *p* < 0.001) in CAT patients undergoing primary SG [26]. Our results suggest that prior operative scarring and increased surgical complexity may exacerbate these risks in conversions, emphasizing the need for meticulous perioperative management including complete blood count checks and close hemodynamic monitoring.

Interestingly, our multivariable analysis did not identify CAT as an independent predictor of complications. This apparent contradiction between the univariate and multivariable analyses suggests that the increased complications in CAT patients are largely attributable to their higher burden of obesity-related conditions rather than anticoagulation therapy itself. In this context, CAT should be interpreted as a surrogate marker for a higher-risk patient profile — characterized by older age, greater comorbidity burden, and higher prevalence of cardiovascular and thromboembolic disease — rather than as an independent risk factor for postoperative adverse events. It is likely that the overlap between factors necessitating CAT (such as cardiovascular disease and thrombotic disorders) and factors increasing surgical risk creates a complex interrelationship that cannot be easily isolated in statistical models. Nevertheless, the higher absolute complication rates remain clinically relevant for perioperative risk assessment and patient counseling [12]. Notably, longer operative time and a history of venous thromboembolism (VTE) emerged as independent predictors of adverse outcomes. Previous studies in primary bariatric surgery have identified VTE history as a significant risk factor, and our findings indicate that this risk persists in conversion procedures [22, 27]. Given the inherent risks associated with revision bariatric surgery, future research should focus on refining perioperative management strategies and optimizing patient selection to mitigate complications in this population.

These findings have important clinical implications. While CAT patients clearly experience higher complication rates, our multivariable analysis demonstrates that this elevated risk is not attributable to anticoagulation therapy per se, but rather to the underlying cardiovascular and thrombotic conditions that necessitate anticoagulation. This distinction is crucial for clinical decision-making: rather than viewing CAT as a contraindication to conversion surgery, surgeons should focus on comprehensive preoperative optimization of obesity-related conditions including diabetes, hypertension, and cardiovascular disease. The association between CAT and adverse outcomes serves as a marker for patients who require enhanced perioperative management, not as an independent risk factor that precludes surgical intervention.

The comparison of outcomes across conversion procedures highlights important safety differences. SG2RYGB was associated with a higher incidence of serious complications, readmissions, and bleeding compared to SG2SADI and SG2BPD. In contrast, Dijkhorst et al. compared SG2RYGB vs. SG2SADI conversions in a smaller cohort that did not consider anticoagulation status and found no significant differences in perioperative outcomes and complications [18]. Therefore, we emphasize the need for special precautions and meticulous perioperative planning in high-risk groups, including individualized anticoagulation protocols and optimization of cardiovascular status, to mitigate complications in high-risk patients undergoing revisional bariatric procedures.

This study offers significant strengths through the MBSAQIP’s large, nationally representative sample with standardized data collection, providing robust statistical power and real-world insights into conversion bariatric surgery for anticoagulated patients—an understudied area. Our findings can inform clinical decision-making and risk stratification for this high-risk population. However, several limitations must be acknowledged. As with all large administrative databases, MBSAQIP has inherent data quality limitations including variable coding accuracy across centers, lack of validation of reported complications, and dependence on voluntary reporting by participating institutions. The retrospective nature of MBSAQIP data limits causal inference and introduces potential confounding. Although multivariable adjustments were applied, unmeasured variables such as institutional protocols, surgeon experience, and patient-specific considerations may have influenced outcomes. Selection bias remains a concern, as surgeons likely tailor procedures based on patient risk, potentially favoring RYGB conversions for higher-risk CAT patients while reserving more complex procedures with malabsorptive components for lower-risk individuals. While the sample size imbalance between CAT (*n* = 935) and non-CAT (*n* = 29,729) groups reflects the true epidemiological distribution, our multivariable regression approach is designed to handle such imbalances and adjust for baseline differences, allowing valid comparison of outcomes between groups. Additionally, the database does not capture critical details such as the indication for anticoagulation, duration of anticoagulation, perioperative bridging protocols, timing of anticoagulation resumption, nor does it differentiate between specific anticoagulant regimens (warfarin, direct oral anticoagulants, low molecular weight heparins) or routes of administration (oral vs. subcutaneous). Different anticoagulants have distinct pharmacokinetic profiles that are differentially affected by altered gastrointestinal anatomy in conversion procedures, potentially impacting both efficacy and bleeding risk postoperatively. The lack of data on bridging therapy strategy (therapeutic vs. prophylactic dosing, agent selection, duration) represents a particularly critical gap, as bridging protocols likely have substantial impact on perioperative bleeding outcomes.

Importantly, MBSAQIP does not distinguish between varying anticoagulation indications that dictate different perioperative management strategies. For example, patients anticoagulated for atrial fibrillation may safely tolerate brief perioperative interruption, while those with mechanical heart valves or recent venous thromboembolism require minimal interruption or therapeutic bridging. These management differences likely have substantial impact on bleeding outcomes but cannot be evaluated with our database. Similarly, the database lacks information on specific perioperative protocols employed (e.g., timing of last preoperative anticoagulant dose, use and type of bridging therapy, postoperative anticoagulation resumption timing), surgeon experience with anticoagulated patients, institutional anticoagulation management pathways, and intraoperative hemostatic techniques. This heterogeneity in anticoagulation indication and perioperative management represents a critical limitation that prevents us from providing specific guidance on optimal protocols for different patient subgroups. MBSAQIP does not capture technical surgical variables including anastomotic technique (stapled vs. handsewn), staple line reinforcement methods, energy device selection, or surgeon-specific factors such as experience with anticoagulated patients, all of which may influence bleeding outcomes.

Finally, the focus on only 30-day outcomes prevents assessing long-term complications and functional outcomes in this high-risk cohort. Despite these limitations, MBSAQIP remains the most comprehensive data source for studying rare clinical scenarios like CAT in conversion bariatric surgery, where prospective trials are impractical. As a contextual consideration, during the study period (2020–2022), perioperative anticoagulation management may have been influenced by COVID-19, as heightened thrombotic awareness led some centers to adopt more aggressive anticoagulation protocols [28–30]. This temporal factor may have contributed to our observed bleeding rates, though the extent of this impact cannot be quantified [22, 28–30]. 

Future research should prioritize prospective studies evaluating perioperative anticoagulation strategies in bariatric surgery, stratified by anticoagulation indication (e.g., atrial fibrillation vs. mechanical valves vs. venous thromboembolism) to account for varying interruption tolerability and bridging requirements. Such studies should compare specific bridging approaches (therapeutic-dose versus prophylactic-dose low molecular weight heparin versus no bridging) and optimal anticoagulation resumption timing in conversion procedures for different clinical scenarios. Developing indication-specific and procedure-specific anticoagulation protocols, validated through multicenter prospective registries with granular data capture, would provide the clinical guidance that large administrative databases cannot deliver. Additionally, investigating the ideal timing for resuming DOACs or warfarin postoperatively, particularly in high-risk conversion cases, is critical. Identifying procedure-specific bleeding risks and refining multidisciplinary management protocols will be essential for improving outcomes in this complex patient population.

## Conclusion

Patients undergoing conversion surgery from SG while on CAT represent a high-risk subset that provides insights into managing complex patients in revisional bariatric surgery. While CAT patients face significantly higher complication rates, increased bleeding risk, and prolonged hospital stays, multivariable analysis revealed that CAT itself is not an independent predictor of adverse outcomes after accounting for obesity-related conditions. The elevated risk observed in this population is driven primarily by the underlying burden of cardiovascular and metabolic disease rather than anticoagulation therapy per se. These findings have broader implications for all high-risk patients undergoing conversion surgery: the presence of multiple obesity-related conditions—whether or not they require anticoagulation—necessitates heightened perioperative vigilance, comprehensive preoperative optimization, and individualized perioperative management strategies. Rather than excluding high-risk patients from surgical treatment, our findings support a risk-stratified approach that emphasizes medical optimization and enhanced perioperative care to improve outcomes in complex patient populations undergoing revisional bariatric procedures.

## Data Availability

No datasets were generated or analysed during the current study.
